# CaAl-Layered Double Hydroxides-Modified Biochar Composites Mitigate the Toxic Effects of Cu and Pb in Soil on Pea Seedlings

**DOI:** 10.3390/ma17112763

**Published:** 2024-06-05

**Authors:** Yuanzheng Wang, Yuhao Cai, Yuxuan Wu, Caiya Yan, Zhi Dang, Hua Yin

**Affiliations:** 1 School of Environment and Energy, South China University of Technology, Guangzhou 510006, Chinayuxuanwu98@163.com (Y.W.);; 2 Key Laboratory of Ministry of Education on Pollution Control and Ecosystem Restoration in Industry Clusters, Guangzhou 510006, China; 3 Guangdong Provincial Key Laboratory of Solid Wastes Pollution Control and Recycling, Guangzhou 510006, China

**Keywords:** mining-contaminated soil, heavy metal contamination, CaAl-layered double hydroxides, pea seedlings

## Abstract

Compound contamination of soil with heavy metals copper (Cu) and lead (Pb) triggered by mining development has become a serious problem. To solve this problem, in this paper, corncob kernel, which is widely available and inexpensive, was used as the raw material of biochar and modified by loading CaAl-layered double hydroxides to synthesize biochar-loaded CaAl-layered double hydroxide composites (CaAl-LDH/BC). After soil remediation experiments, either BC or CaAl-LDH/BC can increase soil pH, and the available phosphorus content and available potassium content in soil. Compared with BC, CaAl-LDH/BC significantly reduced the available content of Cu and Pb in the active state (diethylenetriaminepentaacetic acid extractable state) in the soil, and the passivation rate of Cu and Pb by a 2% dosage of CaAl-LDH/BC reached 47.85% and 37.9%, respectively. CaAl-LDH/BC can significantly enhance the relative abundance of beneficial microorganisms such as *Actinobacteriota*, *Gemmatimonadota*, and *Luteimonas* in the soil, which can help to enhance the tolerance and reduce the enrichment ability of plants to heavy metals. In addition, it was demonstrated by pea seedling (*Pisum sativum* L.) growing experiments that CaAl-LDH/BC increased plant fresh weight, root length, plant height, catalase (CAT) activity, and protein content, which promoted the growth of the plant. Compared with BC, CaAl-LDH/BC significantly reduced the Cu and Pb contents in pea seedlings, in which the Cu and Pb contents in pea seedlings were reduced from 31.97 mg/kg and 74.40 mg/kg to 2.92 mg/kg and 6.67 mg/kg, respectively, after a 2% dosage of CaAl-LDH/BC, which was a reduction of 90.84% and 91.03%, respectively. In conclusion, compared with BC, CaAl-LDH/BC improved soil fertility and thus the plant growth environment, and also more effectively reduced the mobility of heavy metals Cu and Pb in the soil to reduce the enrichment of Cu and Pb by plants.

## 1. Introduction

The processes of mine development, mechanical excavation, blasting, and ore crushing inevitably generate heavy metal-rich dust and slag [1,2,3]. These pollutants can easily spread to the neighboring soils under the action of natural forces such as wind and water flow, which can lead to serious heavy metal pollution problems [4,5,6,7]. In particular, copper (Cu) and lead (Pb), two heavy metal elements commonly found in mining development activities, are particularly significant in terms of their accumulation in the soil environment, which can rapidly reach high concentration levels, posing a serious threat to the balance of the ecosystem and human health [8,9]. Therefore, it is particularly urgent to seek and implement effective remediation methods for soil heavy metal pollution. Among them, in situ, curing/stabilization technology has become an important method for the remediation of heavy metal-contaminated soils due to its cost-effectiveness, simplicity of operation, and significant effect [10].

Biochar, which is characterized by its highly porous nature, large specific surface area, and abundant surface functional groups, is a potential material for the remediation of heavy metal-contaminated soil [11]. The raw material of biochar is natural biomass; it is not only environmentally friendly but also improves soil quality and enhances fertility through the adsorption of nutrients and promotion of microbial activity [12]. However, there are problems with the remediation effectiveness of virgin biochar in the remediation of heavy metal pollution [13]. Therefore, it is particularly important to enhance the effectiveness of biochar materials in the remediation of heavy metal soils.

Layered double hydroxides (LDHs) are multifunctional two ionic layered compounds whose chemical structural formula can be described as M^2+^_1-*x*_M^3+^*_x_*(OH)_2_.A^n−^*_x_*_/n_.zH_2_O, where M^2+^ is metal (II) cation, M^3+^ is metal (III) cation, and A^n−^ is an anion [14,15]. LDHs have a unique anionic intercalation structure, and the metal cations on the layers are flexibly tunable, which can immobilize the heavy metals in the lattice of the LDHs by the ultrastable state mineralization effect. The abundant -OH groups on the LDH laminates make the surface of LDHs rich in binding sites [16]. The structural properties of LDHs have led to their excellent performance in the remediation of heavy metal-contaminated soils [17]. Xu et al. tested the phytotoxicity of LDHs by layered LDHs (S-Mg-LDH and S-Ca-LDH) through the phytotoxicity tests on treated soils and showed that layered LDHs effectively reduced the toxicity of chromium and decreased its bioaccumulation [18]. Although LDHs have high chemical reaction characteristics, they are prone to agglomeration during the reaction process, leading to a decrease in their surface area, which makes their adsorption capacity for heavy metals decrease [19]. Currently, several researchers have addressed this problem by loading layered double hydroxides onto the surface of pristine biochar to improve the pollution remediation effect [17,20]. However, these layered double hydroxides-loaded biochar composites have been mainly applied to heavy metal removal from water, while relatively few studies have been conducted in the remediation of heavy metal-contaminated soils.

In this study, corncob kernel, which is widely available and low-cost, was selected as the raw material of biochar and modified by loading CaAl-layered double hydroxides (CaAl-LDH) to prepare an environmentally friendly composite material of biochar loaded with CaAl-layered double hydroxides (CaAl-LDH/BC) with excellent performance for remediation of Cu- and Pb-polluted mine soils. Through the remediation of Cu and Pb composite-contaminated soil by CaAl-LDH/BC, the changes in soil pH, cation exchange capacity (SOCC), soil organic carbon content (SOC), available phosphorus content (APC), and available potassium content (AKC) of heavy metals after remediation by BC or CaAl-LDH/BC were analyzed, and the effects of soil microbial communities of BC or CaAl-LDH/BC were investigated at a 2% dosage. The effect of CaAl-LDH/BC in mitigating the toxicity of heavy metals on pea seedlings was investigated by a pea seedling pot experiment. In summary, this study aims to provide some theoretical support and review references for the remediation of heavy metal-contaminated soils in mining areas by CaAl-LDH/BC

## 2. Materials and Methods

### 2.1. Chemicals

The main reagents for the experiment are shown in Supplementary Materials Table S1.

### 2.2. CaAl-LDH/BC Preparation

Corncob kernels were rinsed and dried at 60 °C for 48 h, ground and sieved through a 60-mesh sieve (0.25 mm), and then heated to 500 °C at an elevated rate of 5 °C per minute and maintained for 120 min under oxygen-limited conditions to prepare BC. Amounts of 2.5425 g of Ca(NO_3_)_2_·4H_2_O (0.012 mol) and 2.813 g of Al(NO_3_)_3_·9H_2_O (0.006 mol) were accurately weighed and dissolved into a 200 mL beaker containing 100 mL of deionized water to obtain solution A. Solution B was obtained by weighing 5 g of BC into solution A and placing it in the sonicator (JM-10D Jiemeng, Guangzhou, China) for 30 min. A total of 4.0 g of NaOH was dissolved in 50 mL of deionized water to obtain solution C. The beaker containing solution B was placed in an oil bath and the magnetic stirring speed was set at 500 r/min. Solution C was slowly added to solution B at an oil bath temperature of 100 °C and nitrogen gas was introduced for one hour. The beakers were aged in an oven at 60 °C and filtered (filter paper pore size 10 µm) for 10 h. The material was rinsed with deionized water until the pH of the rinsed water did not change. The above material was dried in an oven at a constant temperature of 60 °C for 12 h and passed through a 100-mesh sieve to obtain the material, CaAl-LDH/BC.

### 2.3. Characterization Method

A field emission high-resolution scanning electron microscope (SEM, Sigma500 Zeiss, Oberkochen, Germany), X-ray diffraction (XRD, D8 Advance Bruker, Karlsruhe, Germany), and Fourier transform infrared spectroscopy (FTIR, Nicolet iS10 Thermo Scientific, Waltham, MA, USA) were used to characterize BC and CaAl-LDH/BC.

### 2.4. Experimental Soil Remediation in Mining Areas

Soil samples were collected from the surface of farmland in a mining area in Shaoguan City, Guangdong Province, at depths ranging from 0 to 20 cm, removed from debris, dried naturally, and sieved through a 20-mesh sieve for experimental use.

To study the remediation effect of BC and CaAl-LDH/BC on Cu and Pb composite-contaminated soil, according to the different percentages of BC and CaAl-LDH/BC added to the soil mass, the soil was divided into seven treatment groups, namely, CK (control check), BC 0.5 (0.5% BC + soil), BC 1 (1.0% BC + soil), BC 2 (2.0% BC + soil), LB 0.5 (0.5% CaAl-LDH/BC + soil), LB 1 (1% CaAl-LDH/BC + soil), and LB 2 (2% CaAl-LDH/BC + soil), each with three parallel groups. After 80 d of soil incubation, 60 g of soil from each control group was vacuum freeze-dried, milled, and sieved through a 60-mesh sieve to prepare for the determination of the available content of heavy metals, the distribution of heavy metals, pH, SOCC, SOC, APC, and AKC in the soil. The results are summarized in Table 1 The soil pH was determined by a pH meter after shaking the soil for 30 min at 25 °C in the ratio of 2.5 mL of water to 1 g of soil [21]; the SOCC was measured by centrifuging 3.5 g of soil sample + 50.0 mL of 16.6 mmol/L of hexamine cobalt trichloride (Co(NH_3_)_6_Cl_3_) solution for 60 min at 25 ± 2 °C, and measuring the absorbance at 475 nm after shaking [22]; the soil AKC was generally determined by the ammonium acetate (NH_4_OAc) extraction method [23]; the soil APC was determined according to the ammonium fluoride hydrochloric acid leaching method [24]; the soil SOC was determined by the potassium dichromate oxidation spectrophotometric method [25]; and the available content of Cu and Pb in the soil was determined by flame atomic absorption spectrometry (AA-6880 Shimadzu, Kyoto, Japan) after extraction using the diethylenetriaminepentaacetic acid (DTPA) extraction method [26].

To elucidate the changes in microbial communities in BC- and CaAl-LDH/BC-remediated soils, soil samples (LB2 and BC2) remediated with 2% BC and CaAl-LDH/BC were analyzed by 16S rRNA sequencing in this study (refer to Supplementary Materials Section S2 for specific processes).

### 2.5. Planting Experiments

The remediated soil was planted with pea seedlings and incubated for 30 days at 60% of the maximum water-holding capacity of the field and then sampled for fresh weight, root length, and plant height. The samples were then rinsed with deionized water to remove impurities, and baked at 80 °C until the constant weight and the dry weight were recorded. The samples were crushed in a crusher passed through a 0.25 mm sieve and stored for heavy metal content measurement.

The Catalase (CAT) in plants was measured by the Catalase (CAT) Assay Kit (ADS1027W Jiangsu Addison Biotechnology Co, Ltd., Nanjing, China); the protein content in plants was determined by the BCA Protein Assay Kit (PS1089 Psaitong, Nanjing, China); and the determination of heavy metal content in pea seedlings was performed by flame atomic absorption spectrometry (AA-6880 Shimadzu, Kyoto, Japan) after joint digestion with HClO_4_-HNO_3_ to determine the concentration of Cu and Pb [27].

### 2.6. Chao1 and Shannon Index

The *Chao1* index [28] is a metric used to estimate the species richness in microbial communities. It estimates the number of operational taxonomic units (OTUs) in a community based on the number of species and the number of sequences for certain species, particularly those with only one or two sequences. The *Chao1* index is calculated using the formula:

$$Chao1=Sobs+n_{1}\frac{n_{1}-1}{2(n_{2}+1)}$$

where *Chao1* is the estimated number of OTUs, Sobs is the observed number of OTUs, $n_{1}$ is the number of OTUs with only one sequence, and $n_{2}$ is the number of OTUs with only two sequences.

The *Shannon* index [28], also known as the species diversity index or information entropy index, is a metric used to measure biodiversity. In ecology, the *Shannon* index is widely applied to assess and compare the species diversity in different ecosystems. The *Shannon* index is calculated based on the richness and relative abundance of species. The formula is:

$$H=-\Sigma Pi\times {log}_{2}(Pi)$$

where H is the *Shannon* index, Σ is the summation symbol, and Pi is the relative abundance of each species. A higher *Shannon* index value indicates a higher diversity in the microbial community. This index comprehensively considers both the richness and evenness of species, providing a comprehensive reflection of the diversity level of the biological community.

### 2.7. Statistical Analysis

Statistical analysis and plotting of data in this study were carried out using IBM SPSS Statistics 25 and Origin 2023b software. The labels a, b, c, d, etc. in the figures of this paper indicate the results of the significance analysis.

## 3. Results and Discussion

### 3.1. Material Characterization

**Figure 1:**
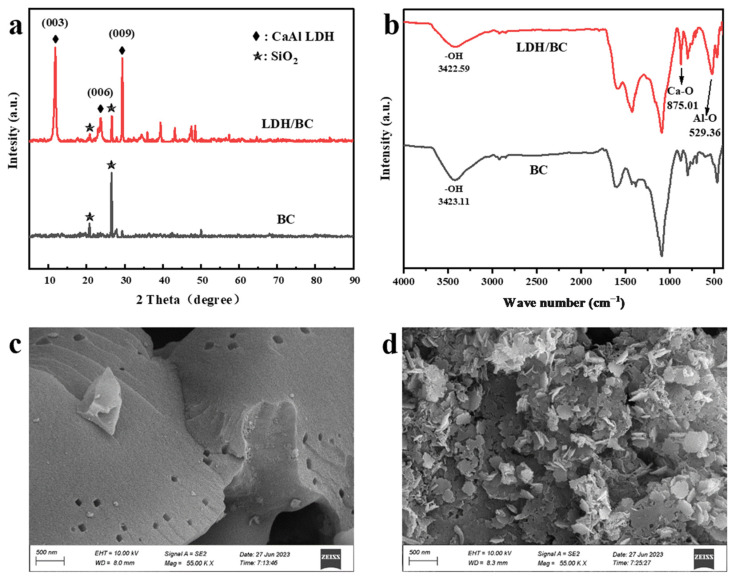
XRD spectra (**a**) and FTIR spectra (**b**) of CaAl-LDH/BC and BC; SEM of BC (**c**) and CaAl-LDH/BC (**d**). (LDH/BC in the figure implies CaAl-LDH/BC).

As shown in the XRD spectra of CaAl-LDH/BC versus BC (Figure 1a), the strong peak at 26.54° corresponds to SiO_2_, which can be attributed to the strong uptake of Si by maize plants [29]. The diffraction peaks of CaAl-LDH/BC are in agreement with those of CaAl-LDH synthesized by Dong Li (PDF 31-0245) [30]. The diffraction peaks present at 11.78°, 23.60°, and 29.38° correspond to the (003), (006), and (009) crystal planes of the layered double hydroxides, indicating that CaAl-LDH was successfully loaded onto BC.

The results of the FTIR spectra of CaAl-LDH/BC are shown in Figure 1b. The characteristic peaks at 875.01 cm^−1^ and 525.36 cm^−1^ correspond to Ca-O and Al-O groups, respectively, which further confirmed the loading of CaAl-LDH onto the surface of BC [31,32]. Moreover, compared to BC, CaAl-LDH/BC corresponds to a smaller hydroxyl characteristic peak area near 3422.59 cm^−1^, which is a result of the loading of the CaAl-LDH leading to the weakening of the reducing functional groups of the material [33]. The surface morphologies of BC and CaAL-LDH/BC are shown in Figure 1c, and Figure 1d, respectively. In comparison to BC, the composites have a dense accumulation of lamellar CaAl-LDH adherence on the surface of the biochar, which provides more adsorption sites for heavy metal adsorption [34].

### 3.2. Effect of CaAl-LDH/BC Composites on Soil Physicochemical Properties

**Figure 2:**
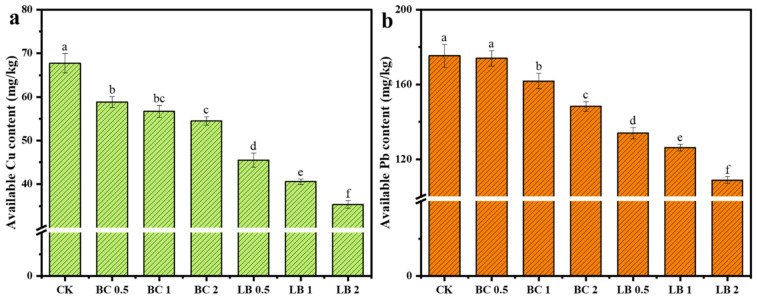
Effect of BC and CaAl-LDH/BC on Cu (**a**) and Pb (**b**) effective state concentrations. (The a, b, c, d, e, and f near the error bars represent significant differences according to the Student’s *t*-test [*p* < 0.5]).

As shown in Table 1, BC and CaAl-LDH/BC could increase the SOC, AKC, and APC in the soil, and compared with BC, CaAl-LDH/BC could better increase the soil pH and SOCC, which indicated that CaAl-LDH/BC could effectively improve the soil physicochemical properties and enhance the soil fertility. As shown in Figure 2, BC and CaAl-LDH/BC reduced the available Cu content from 67.7 mg/kg to 54.5–58.8 mg/kg and 35.3–45.5 mg/kg, respectively, with the lowest available Cu content of 35.3 mg/kg at 2.0% of CaAl-LDH/BC, which was 47.85%. BC and CaAl-LDH/BC reduced the available content of Pb in soil from 175.3 mg/kg to 148.2~174.0 mg/kg and 108.8~133.9 mg/kg, respectively, and the available content of Pb in soil was the lowest when the dosage of CaAl-LDH/BC was 2.0%, which decreased by 37.95% to 108.8 mg/kg. The passivation effect was significantly better than that of the BC treatment group. This indicates that relative to BC, the CaAl-LDH/BC composite has a stronger passivation capacity for Cu and Pb in soil and can effectively reduce the available content of Cu and Pb in soil. In addition, as shown in Table 2, CaAl-LDH/BC also had an excellent performance compared to other restorative materials.

### 3.3. Effects of CaAl-LDH/BC on the Diversity of Soil Microbial Communities

To assess the species diversity of the total soil microbial community, the microbiota *Chao1* index, *Shannon* index, and Coverage indices were analyzed. As shown in Table 3, the Coverage of the CK, BC 2, and LB 2 groups exceeded 0.999, which indicates that the microbial sequencing results of the samples are very reliable. The *Chao1* index and *Shannon* index values after treatment with a 2% dosage of BC or CaAl-LDH/BC showed an increase. The average *Chao1* index and average *Shannon* index values of the microbial community in the BC treatment group increased from 417.94 and 5.09 in the CK group to 562.20 and 6.19, which indicated that the addition of biochar was beneficial to the increase in microbial community diversity and richness in the soil, which is consistent with [39] and the fact that the herbaceous biochar can increase soil porosity due to its highly porous nature and play a role in protecting microorganisms, thus promoting the growth and diversity of microbial biomass. The average *Chao1* index and average *Shannon* index values of the CaAl-LDH/BC treatment group reached 811.56 and 6.45, and the *Chao1* index in particular was much higher than that of the CK group (417.94) and that of the BC treatment group (562.20). This phenomenon may be attributed to the fact that the CaAl-LDH/BC can reduce the available content of heavy metals (Table 1) and increase the APC and AKC in the soil (Table 1) more efficiently than BC, thus providing better environmental conditions for the diversity of soil microbial communities and significantly contributing to the growth of species number and the equalization of species distribution. This is consistent with Huang et al.’s finding that the increase in nutrients such as nitrogen, phosphorus, and potassium in the soil was favorable to the increase in species diversity of soil microbial communities [40].

### 3.4. Effects of CaAl-LDH/BC on the Structural Composition of Soil Microbial Communities

To deeply investigate the specific effects on soil microbial communities produced by BC and CaAl-LDH/BC when remediating Cu- and Pb-contaminated soils at 2% addition, the changes in the relative abundance of microbial communities at the phylum and genus levels after the treatment of these two remediation materials were analyzed in this paper.

**Figure 3:**
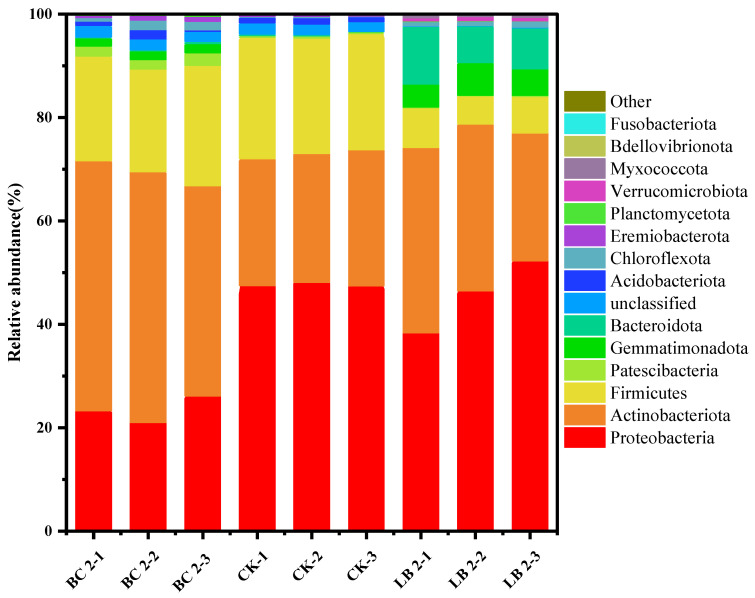
Effects of BC 2 and LB 2 on microbial communities at the gate level.

As shown in Figure 3, the microbial communities in the soil of both the CK and BC 2 groups were mainly composed of *Proteobacteria*, *Actinobacteria*, and *Firmicutes* at the phylum level. This suggests that after BC remediation, the soil microbial community did not show significant changes in its major composition at the gate level and still maintained a similar microbial population structure to that of the unremediated soil. This is in line with previous reports that microorganisms such as Proteobacteria, *Actinobacteriota*, and *Firmicutes* have a strong tolerance to heavy metal pollutants [41]. Among them, the average relative abundance of *Actinobacteriota* increased from 25.27% to 45.87%, and Cui et al. [42] found that *Actinobacteriota* reduces the heavy metal content of plants, which is one of the reasons why BC can reduce the enrichment of heavy metals by plants. As for the main phyla in the soil microbial community of the LB2 group, in addition to the three phyla mentioned above, the presence of *Gemmatimonadota* and *Bacteroidota* suggests that CaAl-LDH/BC favors an increase in the number of dominant species of microorganisms in the soil, which once again proves that CaAl-LDH/BC favors an increase in the richness and diversity of the soil microbial community. After treatment with 2% dosing of CaAl-LDH/BC, the soil microbial community showed the following changes at the gate level: The average relative abundance of actinomycetes increased from 25.58% to 30.95%, contributing to the reduction in heavy metal enrichment by plants [42]. Previous studies have shown that *Bacteroidota* in soil has a strong degradation capacity for plant residues such as straw, and its average relative abundance increased from 0.2% to 8.72%, which can effectively promote carbon and nitrogen cycling in the soil, which in turn is conducive to the maintenance of the nutrient balance of the soil ecosystem [43]. The average relative abundance of *Gemmatimonadota* was elevated from 0.009% to 5.30%, and Oshiki et al. found that *Gemmatimonadota* could effectively reduce the emission of N_2_O from the soil, which is conducive to increasing the N content of the soil and contributing to the fertility enhancement of the soil [44].

This suggests that CaAl-LDH/BC, relative to BC, can increase the average relative abundance not only of *Actinobacteriota* but also of *Gemmatimonadota* and *Bacteroidota*, suggesting that CaAl-LDH/BC can be more effective in increasing the diversity of microbial communities.

**Figure 4:**
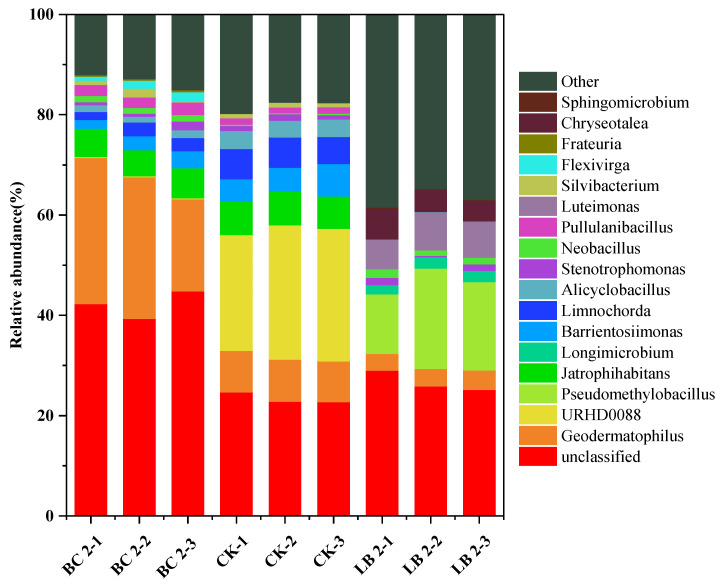
Effects of BC 2 and LB 2 on microbial communities at the genus level.

As can be seen in Figure 4, the soil microorganisms in each group of BC2, CK, and LB2, at the genus level, differed greatly in composition, indicating that both BC and CaAl-LDH/BC significantly altered the composition of the microbial community in the soil at the genus level. The average relative abundance of *Geodermatophilus* increased from 8.27% to 25.24% in the CK group in the soil remediated with 2% BC. Some studies have reported that *Geodermatophilus* can be found in the rhizosphere of nickel-hyperaccumulating plants [45], which may favor the enrichment of heavy metals in soil by plants. *Pseudomethylobacillus*, *Luteimonas, Chryseotalea,* and *Longimicrobium* appeared as the dominant genera at the gate level in the soil remediated with a 2% dosage of LDH/BC, where *Pseudomethylobacillus* and *Chryseotalea* increased the average relative abundance from 0.0038% and 0% to 16.47% and 6.8%, respectively, and it has been reported that both of them are found in freshwater water and their occurrence in the soil may imply the improvement in the soil environment [46,47]. Based on previous reports [48], *Luteimonas* is one of the core genera in composting that promotes the process of nitrogen transformation and thus reduces nitrogen loss, indicating that an increase in the average relative abundance of *Luteimonas* from 0.0004% to 6.80% is beneficial for improving the nitrogen fixation capacity of the soil. The average relative abundance of *Longimicrobium* increased from 0% to 2.14%, and available studies have shown that *Longimicrobium* can promote plant tolerance to heavy metal and salt stress, nutrient uptake, and plant growth [49].

As a result, CaAl-LDH/BC can more effectively increase the diversity of soil microbial communities relative to BC, which in turn better improves soil fertility and promotes plant tolerance to heavy metals.

### 3.5. Changes in the Growth of Pea Seedlings

To assess the effects of BC and CaAl-LDH/BC on plant growth after remediation of contaminated soil in the mining area, the root length, plant height, and fresh weight of pea seedlings after 30 d of growth were analyzed in this paper.

**Figure 5:**
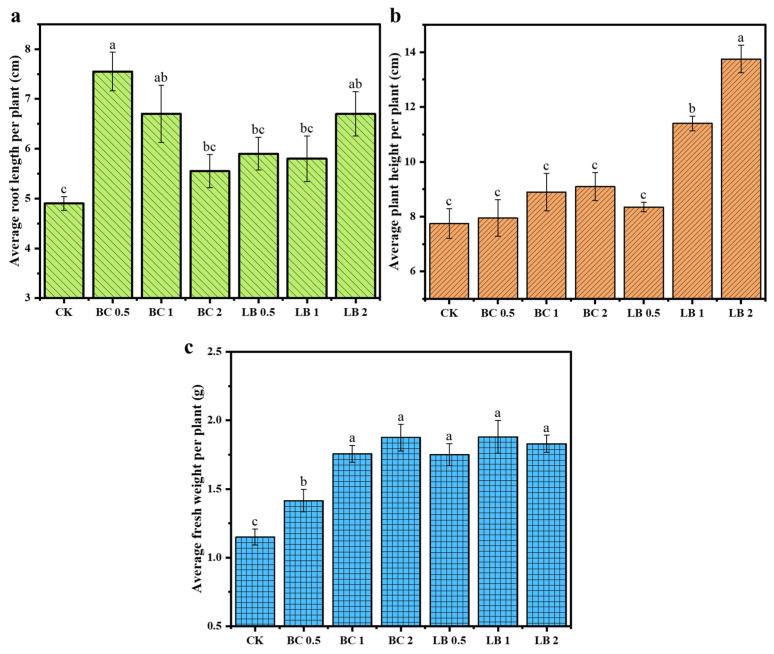
Average root length (**a**), average plant height (**b**), and average fresh weight (**c**) of pea seedlings in blank and treated soils. (The a, b, and c near the error bars represent significant differences according to the Student’s *t*-test [*p* < 0.5]).

As shown in Figure 5, the average root length, plant height, and fresh weight of pea seedlings in the CK group were 4.9 cm, 7.77 cm, and 1.14 g, respectively, while those of pea seedlings in the BC group ranged from 5.55 to 7.55 cm, 7.93 to 9.10 cm, and 1.41 to 1.87 g, respectively. The BC0.5 group had the longest average root length of 7.55 cm; the BC2 group had the largest average plant height and fresh weight of 9.10 cm and 1.87 g, respectively. The average root length, plant height, and fresh weight of pea seedlings in the LB group were 5.9–6.7 cm, 8.35–13.73 cm, and 1.75–1.88 g, respectively. The average root length of the LB2 group was the longest at 6.7 cm; the average plant height of the LB2 group was the longest at 13.73 cm; and the average fresh weight of the LB 1 group was the largest at 1.88 g. This indicated that BC and CaAl-LDH/BC increased the root length and fresh weight of pea seedlings, which was favorable to improving the plant’s ability to absorb nutrients and water from the soil. Compared with BC, CaAl-LDH/BC was more favorable to the growth of plant height of pea seedlings, which indicated that CaAl-LDH/BC had a better promotion effect on the growth and development of pea seedlings.

### 3.6. Changes in CAT Activity and Protein Content in Pea Seedlings

To further investigate the effects of BC and CaAl-LDH/BC on the metabolic activity and growth of plants after remediation of contaminated soils in the mining area, the CAT activity and protein contents of pea seedlings incubated for 30 d were examined.

**Figure 6:**
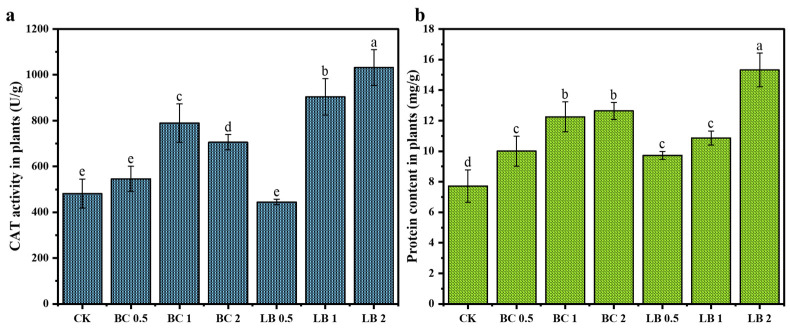
CAT activity (**a**) and protein content (**b**) of pea seedlings in soil. (The a, b, c, d, and e near the error bars represent significant differences according to the Student’s *t*-test [*p* < 0.5]).

The results of CAT activity in pea seedlings are shown in Figure 6a. The CAT activity in pea seedlings of the CK group was 481.65 U/mg, and that in pea seedlings of the BC group ranged from 545.87 to 788.99 U/mg, with the highest activity in the BC 1 group (BC dosage of 1%), which was 788.99 U/mg. The CAT activity in pea seedlings of the LB group ranged from 444.95 to 1032.11 U/mg, with the highest activity of 1032.11 U/mg in the LB 2 group, and the CAT activity tended to increase with the dosage of CaAl-LDH/BC. It indicated that compared to BC, CaAl-LDH/BC could increase CAT activity in pea seedlings more effectively and mitigate the toxic effects of heavy metals on pea seedlings. As shown in Figure 6b, the protein content of pea seedlings in the control group (CK) was 7.71 mg/g, and the protein content of pea seedlings in the BC group ranged from 10.00 to 12.64 mg/g, with the highest protein content being 12.64 mg/g in the BC 2 group. The protein content of pea seedlings in the LB group ranged from 9.72 to 15.31 mg/g, with the LB 2 group having the highest at 15.31 mg/g, and these data indicated a positive correlation between protein content and the addition of CaAl-LDH/BC. It indicated that CaAl-LDH/BC increased the biosynthesis capacity of plants, which may be related to the ability of CaAl-LDH/BC complexes to improve the fertility nutrients of the soil.

### 3.7. Changes in Cu and Pb Content in Pea Seedlings

To further investigate the effects of BC and CaAl-LDH/BC on the remediation of contaminated soil in the mining area and the Cu and Pb contents in plants, the Cu and Pb contents in pea seedlings incubated for 30 days were examined in this paper.

**Figure 7:**
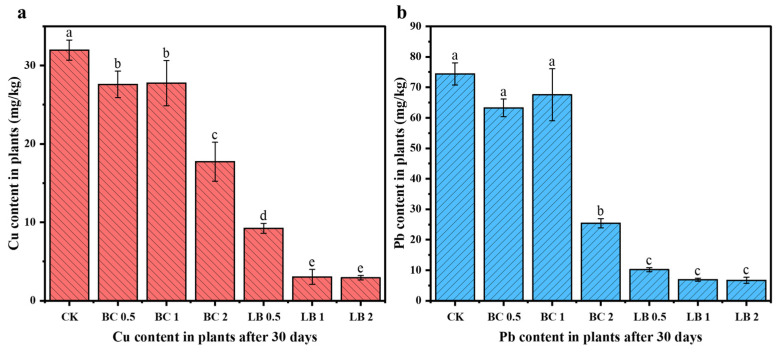
Cu content (**a**) and Pb content (**b**) of pea seedlings in treated soils. (The a, b, c, d and e near the error bars represent significant differences according to the Student’s *t*-test [*p* < 0.5]).

As shown in Figure 7, the Cu and Pb contents of pea seedlings in BC group decreased from 31.97 mg/kg and 74.40 mg/kg to 17.59–27.59 mg/kg and 25.40–63.23 mg/kg, respectively, with the lowest Cu and Pb contents of pea seedlings occurring in the BC 2 group at 17.59 mg/kg and 25.40 mg/kg, respectively. In the LB group, the Cu and Pb contents of pea seedlings ranged from 2.92 to 9.23 mg/kg and 6.67 to 10.18 mg/kg, respectively. Among them, the pea seedlings in the LB 2 group had the lowest Cu and Pb contents of 2.92 mg/kg and 6.67 mg/kg, which were reduced by 90.84% and 91.03%, respectively, when compared with pea seedlings in the CK group. Relative to the CK group, the Cu and Pb contents of pea seedlings in each treatment group showed a decrease, and the Cu and Pb contents of pea seedlings decreased with the increase in material dosing, which again proved the passivating effect of BC and CaAl-LDH/BC on Cu and Pb in the polluted soil of the mining area.

## 4. Conclusions

In this study, a novel biocarbon material, CaAl-LDH/BC, was prepared, which effectively reduced the content of Cu and Pb in the active state of the soil in the mining area, improved the structure of the soil microbial community composition, promoted the growth of plants in the soil, and reduced the enrichment of Cu and Pb by plants. The main conclusions are as follows:

1. CaAl-LDH/BC could increase the content of SOC, AKC, and APC in the soil, and compared with BC, CaAl-LDH/BC could better increase the soil pH and cation exchange, which indicated that CaAl-LDH/BC could more effectively promote soil improvement and fertility enhancement. Furthermore, CaAl-LDH/BC significantly reduced the available content of Cu and Pb in the soil, of which the 2% dosage of CaAl-LDH/BC showed the best effect, and its passivation of Cu and Pb in soil reached 47.85% and 37.95%, respectively.
2. Based on the results of 16S rRNA gene sequencing, the *Chao1* index and *Shannon* index values of microbial communities in CaAl-LDH/BC-restored soils were significantly increased compared with those of BC, indicating that CaAl-LDH/BC had a significant effect on enhancing the diversity and abundance of soil microorganisms. CaAl-LDH/BC could significantly enhance the average relative abundance of beneficial microorganisms such as *Actinobacteriota*, *Gemmatimonadota*, *Luteimonas*, and *Longimicrobium* in the soil, which could help to enhance the tolerance of plants to heavy metals, reduce the enrichment capacity of plants for heavy metals, and enhance the nitrogen fixation capacity of the soil, It indicated that CaAl-LDH/BC had a positive effect on improving the soil microbial environment and inducing microbial communities to reduce the damage of heavy metals to plants.
3. CaAl-LDH/BC can increase the fresh weight, root length, and plant height of pea seedlings, promote the growth of pea seedlings, reduce the content of Cu and Pb in pea seedlings, and enhance the CAT activity and protein content in pea seedlings. The lowest Cu and Pb contents in pea seedlings were 2.92 mg/kg and 6.67 mg/kg, which were reduced by 90.84% and 91.03%, respectively, after 2% CaAl-LDH/BC treatment.

This indicates that, relative to BC, CaAl-LDH/BC not only effectively and significantly improves the soil environment and plant growth conditions and promotes crop growth, but also further immobilizes Cu and Pb in the soil and reduces the degree of Cu and Pb enrichment in pea seedlings, showing its excellent ability in improving the soil environment and separating Cu and Pb. This paper provides theoretical support and reference for CaAl-LDH/BC in the remediation of heavy metal-contaminated soil.

## Figures and Tables

**Table 1:** Changes in physicochemical properties of BC- and CaAl-LDH/BC-remediated soils 80 d after remediation.

	pH	AKC	APC	SOCC	SOC
		mg/kg	mg/kg	mmol/kg	mg/kg
CK	4.47	327.63	30.88	32.59	11.75
BC 0.5	4.54	346.50	31.45	37.43	15.73
BC 1	4.66	376.05	31.63	39.54	18.66
BC 2	4.92	402.00	31.73	36.59	25.92
LB 0.5	5.30	343.03	34.50	38.71	14.80
LB 1	5.75	384.16	34.71	49.75	17.22
LB 2	6.33	444.11	35.72	62.91	24.65

**Table 2:** Comparison of the performance of CaAl-LDH/BC with other restorative materials.

Feed Material	Dosage	Soil Heavy Metal Contaminants	Processing Times	Raw Soil Heavy Metal Content	Reference
Cornstalk biochar	2%	The available contents of Cd, Cu, Zn, and Pb were reduced by 23.8%, 11.9%, 5.27%, and 14.3%, respectively.	28 d	The available contents of Cd, Cu, Zn, and Pb were 3.2, 17, 108, and 145 mg/kg, respectively.	[35]
Miscanthus × giganteus biochar	1%	CaCl_2_-extractable Cd, Zn, and Pb concentrations were reduced by 14%, 15%, and 29%, respectively.	84 d	CaCl_2_-extractable Cd, Zn, and Pb concentrations were 24.0, 2980, and 3110 mg/kg.	[36]
Nano-Montmorillonite	1%	Cd available content was reduced by 5.59%.	60 d	Cd available content was 2.51 mg/kg.	[37]
Red mud/Apatite/Red mud + Apatite	5%/5%/2.5% + 2.5%	Bioavailable and potentially bioavailable fraction of lead reduced from 92.1% to 80.2%, 86.4%, and 82.2%, respectively.	120 d	The total content of Pb was 44.2 mg/kg.	[38]
CaAl-LDH/BC	2%	The available contents of Pb and Cu were reduced by 37.95% and 47.85%.	80 d	The available contents of Pb and Cu were 175.3 and 67.7 mg/kg.	This paper

**Table 3:** Effects of BC and CaAl-LDH/BC on the α diversity of microbial communities.

	*Chao1*	*ACE*	*Shannon*	*Simpson*	Coverage
BC 2-1	515.447	518.8916	6.072507	0.964053	0.999863
BC 2-2	592.8255	594.9367	6.208726	0.967411	0.999871
BC 2-3	571.3871	572.7808	6.300023	0.971114	0.999887
CK-1	402.8966	405.1257	5.154584	0.918712	0.999919
CK-2	402.3636	404.6076	5.052613	0.90576	0.999902
CK-3	448.561	452.0917	5.049857	0.906209	0.999784
LB 2-1	817.1224	820.1697	6.564774	0.970319	0.999827
LB 2-2	808.5941	819.0188	6.224698	0.953588	0.999676
LB 2-3	808.9655	807.51	6.564133	0.963213	0.999857

## Data Availability

The raw data supporting the conclusions of this article will be made available by the authors on request.

## Supplementary Materials

The following supporting information can be downloaded at: https://www.mdpi.com/article/10.3390/ma17112763/s1, Table S1: The main experimental drugs; Section S2: 16S rRNA sequencing specific processes.

## References

[1] Baran H.A., Nalbantcilar M.T., Koktan N. (2023). Pollution and Health Risk Assessment of Heavy Metals in Waters around Mine Sites of Elazig (Eastern Turkey). J. Mt. Sci..

[2] Fazle Bari A.S.M., Lamb D., MacFarlane G.R., Rahman M.M. (2022). Soil Washing of Arsenic from Mixed Contaminated Abandoned Mine Soils and Fate of Arsenic after Washing. Chemosphere.

[3] Kavehei A., Hose G.C., Chariton A.A., Gore D.B. (2021). Application of Environmental DNA for Assessment of Contamination Downstream of a Legacy Base Metal Mine. J. Hazard. Mater..

[4] Mandal J., Bakare W.A., Rahman M.M., Rahman M.A., Siddique A.B., Oku E., Wood M.D., Hutchinson S.M., Mondal D. (2022). Varietal Differences Influence Arsenic and Lead Contamination of Rice Grown in Mining Impacted Agricultural Fields of Zamfara State, Nigeria. Chemosphere.

[5] Igwe O., Una C.O., Abu E., Adepehin E.J. (2017). Environmental Risk Assessment of Lead–Zinc Mining: A Case Study of Adudu Metallogenic Province, Middle Benue Trough, Nigeria. Environ. Monit. Assess..

[6] Ayari J., Barbieri M., Agnan Y., Sellami A., Braham A., Dhaha F., Charef A. (2021). Trace Element Contamination in the Mine-Affected Stream Sediments of Oued Rarai in North-Western Tunisia: A River Basin Scale Assessment. Environ. Geochem. Health.

[7] Byrne P., Wood P.J., Reid I. (2012). The Impairment of River Systems by Metal Mine Contamination: A Review Including Remediation Options. Crit. Rev. Environ. Sci. Technol..

[8] Garg R., Garg R., Khan M.A., Bansal M., Garg V.K. (2023). Utilization of Biosynthesized Silica-Supported Iron Oxide Nanocomposites for the Adsorptive Removal of Heavy Metal Ions from Aqueous Solutions. Environ. Sci. Pollut. Res..

[9] Petit J.C.J., Maggi P., Pirard C., Charlier C., Ruttens A., Liénard A., Colinet G., Remy S. (2022). Human Biomonitoring Survey (Pb, Cd, As, Cu, Zn, Mo) for Urban Gardeners Exposed to Metal Contaminated Soils. Environ. Pollut..

[10] Azhar U., Ahmad H., Shafqat H., Babar M., Shahzad Munir H.M., Sagir M., Arif M., Hassan A., Rachmadona N., Rajendran S. (2022). Remediation Techniques for Elimination of Heavy Metal Pollutants from Soil: A Review. Environ. Res..

[11] Liang X., Su Y., Wang X., Liang C., Tang C., Wei J., Liu K., Ma J., Yu F., Li Y. (2023). Insights into the Heavy Metal Adsorption and Immobilization Mechanisms of CaFe-Layered Double Hydroxide Corn Straw Biochar: Synthesis and Application in a Combined Heavy Metal-Contaminated Environment. Chemosphere.

[12] Wang Y., Liu Y., Zhan W., Zheng K., Wang J., Zhang C., Chen R. (2020). Stabilization of Heavy Metal-Contaminated Soils by Biochar: Challenges and Recommendations. Sci. Total Environ..

[13] Lee H.-S., Shin H.-S. (2021). Competitive Adsorption of Heavy Metals onto Modified Biochars: Comparison of Biochar Properties and Modification Methods. J. Environ. Manag..

[14] Jijoe P.S., Yashas S.R., Shivaraju H.P. (2021). Fundamentals, Synthesis, Characterization and Environmental Applications of Layered Double Hydroxides: A Review. Environ. Chem. Lett..

[15] Mishra G., Dash B., Pandey S. (2018). Layered Double Hydroxides: A Brief Review from Fundamentals to Application as Evolving Biomaterials. Appl. Clay Sci..

[16] Zhang Q., Zhang G., Huang Y., He S., Li Y., Jin L., Han J. (2024). Surface-Modified LDH Nanosheets with High Dispersibility in Oil for Friction and Wear Reduction. ACS Appl. Mater. Interfaces.

[17] Lyu P., Li L., Huang J., Ye J., Zhu C., Xie J., Wang Z., Kang M., Yan A. (2023). Enhancing Sorption of Layered Double Hydroxide-Based Magnetic Biochar for Arsenic and Cadmium through Optimized Preparation Protocols. Bioresour. Technol..

[18] Xu S., Zhang L., Zhao J., Cheng J., Yu Q., Zhang S., Zhao J., Qiu X. (2020). Remediation of Chromium-Contaminated Soil Using Delaminated Layered Double Hydroxides with Different Divalent Metals. Chemosphere.

[19] Huang W.-H., Chang Y.-J., Lee D.-J. (2024). Layered Double Hydroxide Loaded Pinecone Biochar as Adsorbent for Heavy Metals and Phosphate Ion Removal from Water. Bioresour. Technol..

[20] Veselska V., Sillerova H., Hudcova B., Ratie G., Lacina P., Lalinska-Volekova B., Trakal L., Sottnik P., Jurkovic L., Pohorely M. (2022). Innovative in Situ Remediation of Mine Waters Using a Layered Double Hydroxide-Biochar Composite. J. Hazard. Mater..

[21] Puissant J., Jones B., Goodall T., Mang D., Blaud A., Gweon H.S., Malik A., Jones D.L., Clark I.M., Hirsch P.R. (2019). The pH Optimum of Soil Exoenzymes Adapt to Long Term Changes in Soil pH. Soil Biol. Biochem..

[22] Aran D., Maul A., Masfaraud J.-F. (2008). A Spectrophotometric Measurement of Soil Cation Exchange Capacity Based on Cobaltihexamine Chloride Absorbance. Comptes Rendus Geosci..

[23] Adhikari S., Moon E., Timms W. (2024). Identifying Biochar Production Variables to Maximise Exchangeable Cations and Increase Nutrient Availability in Soils. J. Clean. Prod..

[24] Gao S., Zhang S., Yuan L., Li Y., Zhao L., Wen Y., Xu J., Hu S., Zhao B. (2023). Effects of Humic Acid–Enhanced Phosphate Fertilizer on Wheat Yield, Phosphorus Uptake, and Soil Available Phosphorus Content. Crop Sci..

[25] Liu T., Guo L., Cao C., Tan W., Li C. (2021). Long-Term Rice-Oilseed Rape Rotation Increases Soil Organic Carbon by Improving Functional Groups of Soil Organic Matter. Agric. Ecosyst. Environ..

[26] Dai J., Becquer T., Henri Rouiller J., Reversat G., Bernhard-Reversat F., Nahmani J., Lavelle P. (2004). Heavy Metal Accumulation by Two Earthworm Species and Its Relationship to Total and DTPA-Extractable Metals in Soils. Soil Biol. Biochem..

[27] Bonanno G., Borg J.A., Di Martino V. (2017). Levels of Heavy Metals in Wetland and Marine Vascular Plants and Their Biomonitoring Potential: A Comparative Assessment. Sci. Total Environ..

[28] Han S., Li X., Luo X., Wen S., Chen W., Huang Q. (2018). Nitrite-Oxidizing Bacteria Community Composition and Diversity Are Influenced by Fertilizer Regimes, but Are Independent of the Soil Aggregate in Acidic Subtropical Red Soil. Front. Microbiol..

[29] Peng Y., Sun Y., Sun R., Zhou Y., Tsang D.C.W., Chen Q. (2019). Optimizing the Synthesis of Fe/Al (Hydr)Oxides-Biochars to Maximize Phosphate Removal via Response Surface Model. J. Clean. Prod..

[30] Li D., Yan W., Guo X., Tian Q., Xu Z., Zhu L. (2020). Removal of Selenium from Caustic Solution by Adsorption with CaAl Layered Double Hydroxides. Hydrometallurgy.

[31] Wang J., Kang Y., Duan H., Zhou Y., Li H., Chen S., Tian F., Li L., Drosos M., Dong C. (2022). Remediation of Cd^2+^ in Aqueous Systems by Alkali-Modified (Ca) Biochar and Quantitative Analysis of Its Mechanism. Arab. J. Chem..

[32] He X., Jiang J., Hong Z., Pan X., Dong Y., Xu R. (2020). Effect of Aluminum Modification of Rice Straw–Based Biochar on Arsenate Adsorption. J. Soils Sediments.

[33] Wang F., Jin L., Guo C., Min L., Zhang P., Sun H., Zhu H., Zhang C. (2021). Enhanced Heavy Metals Sorption by Modified Biochars Derived from Pig Manure. Sci. Total Environ..

[34] Sun Z., Wang Y., Liu T., Kong X., Pan T., Zhang F., Lei X., Duan X. (2023). Super-Stable Mineralization of Cu, Cd, Zn and Pb by CaAl-Layered Double Hydroxide: Performance, Mechanism, and Large-Scale Application in Agriculture Soil Remediation. J. Hazard. Mater..

[35] Huang C., Wang W., Yue S., Adeel M., Qiao Y. (2020). Role of Biochar and *Eisenia fetida* on Metal Bioavailability and Biochar Effects on Earthworm Fitness. Environ. Pollut..

[36] Houben D., Evrard L., Sonnet P. (2013). Beneficial Effects of Biochar Application to Contaminated Soils on the Bioavailability of Cd, Pb and Zn and the Biomass Production of Rapeseed (*Brassica napus* L.). Biomass Bioenergy.

[37] Qin C., Yuan X., Xiong T., Tan Y.Z., Wang H. (2020). Physicochemical Properties, Metal Availability and Bacterial Community Structure in Heavy Metal-Polluted Soil Remediated by Montmorillonite-Based Amendments. Chemosphere.

[38] Shin W., Kim Y.-K. (2016). Stabilization of Heavy Metal Contaminated Marine Sediments with Red Mud and Apatite Composite. J. Soils Sediments.

[39] Singh H., Northup B.K., Rice C.W., Prasad P.V.V. (2022). Biochar Applications Influence Soil Physical and Chemical Properties, Microbial Diversity, and Crop Productivity: A Meta-Analysis. Biochar.

[40] Huang H., Liu H., Zhang R., Chen Y., Lei L., Qiu C., Xu H. (2022). Effect of Slow-Released Biomass Alkaline Amendments Oyster Shell on Microecology in Acidic Heavy Metal Contaminated Paddy Soils. J. Environ. Manag..

[41] Luo J., Tao Q., Jupa R., Liu Y., Wu K., Song Y., Li J., Huang Y., Zou L., Liang Y. (2019). Role of Vertical Transmission of Shoot Endophytes in Root-Associated Microbiome Assembly and Heavy Metal Hyperaccumulation in Sedum Alfredii. Environ. Sci. Technol..

[42] Cui H., Liu L.-L., Dai J.-R., Yu X.-N., Guo X., Yi S.-J., Zhou D.-Y., Guo W.-H., Du N. (2018). Bacterial Community Shaped by Heavy Metals and Contributing to Health Risks in Cornfields. Ecotoxicol. Environ. Saf..

[43] Huang J., Gao K., Yang L., Lu Y. (2023). Successional Action of *Bacteroidota* and *Firmicutes* in Decomposing Straw Polymers in a Paddy Soil. Environ. Microbiome.

[44] Oshiki M., Toyama Y., Suenaga T., Terada A., Kasahara Y., Yamaguchi T., Araki N. (2022). N_2_O Reduction by Gemmatimonas Aurantiaca and Potential Involvement of Gemmatimonadetes Bacteria in N_2_O Reduction in Agricultural Soils. Microbes Environ..

[45] Touceda-González M., Kidd P.S., Smalla K., Prieto-Fernández A. (2018). Bacterial Communities in the Rhizosphere of Different Populations of the Ni-Hyperaccumulator Alyssum Serpyllifolium and the Metal-Excluder Dactylis Glomerata Growing in Ultramafic Soils. Plant Soil.

[46] Sheu C., Cai C.-Y., Sheu S.-Y., Li Z.-H., Chen W.-M. (2019). Pseudomethylobacillus Aquaticus Gen. Nov., Sp. Nov., a New Member of the Family Methylophilaceae Isolated from an Artificial Reservoir. Int. J. Syst. Evol. Microbiol..

[47] Pitt A., Lienbacher S., Schmidt J., Neumann-Schaal M., Wolf J., Hahn M.W. (2024). Description of a New Freshwater Bacterium Aquirufa Regiilacus Sp. Nov., Classification of the Genera Aquirufa, Arundinibacter, Sandaracinomonas, and Tellurirhabdus to the Family Spirosomataceae, Classification of the Genus Chryseotalea to the Family Fulvivirgaceae and Litoribacter to the Family Cyclobacteriaceae, as Well as Classification of Litoribacter Alkaliphilus as a Later Heterotypic Synonym of Litoribacter Ruber. Arch. Microbiol..

[48] Liu N., Liu Z., Wang K., Zhao J., Fang J., Liu G., Yao H., Pan J. (2024). Comparison Analysis of Microbial Agent and Different Compost Material on Microbial Community and Nitrogen Transformation Genes Dynamic Changes during Pig Manure Compost. Bioresour. Technol..

[49] Li X., Zhang Z., Luo J., Cui X., Xu J., Hou Y., Hao B., Guo W. (2022). Arbuscular Mycorrhizal Fungi and Nitrilotriacetic Acid Regulated *Suaeda Salsa* Growth in Cd-Contaminated Saline Soil by Driving Rhizosphere Bacterial Assemblages. Environ. Exp. Bot..

