# Supplementary material for: CaAl-Layered Double Hydroxides-Modified Biochar Composites Mitigate the Toxic Effects of Cu and Pb in Soil on Pea Seedlings
Source: Materials (Basel). 2024 Jun 5;17(11):2763. doi: 10.3390/ma17112763 (PMC11173730; doi:10.3390/ma17112763)
Supplement: Supplementary file 1 [file materials-17-02763-s001.zip › materials-2993781-supplementary.pdf]

Supplementary Materials

CaAl-Layered Double Hydroxides-Modified Biochar Composites Mitigate the Toxic Effects of Cu and Pb in Soil on Pea Seedlings

Yuanzheng Wang [1], Yuhao Cai [1], Yuxuan Wu [1], Caiya Yan [1], Zhi Dang [1,2,3] and Hua Yin [1,2,3,*]

[1] School of Environment and Energy, South China University of Technology, Guangzhou 510006, China; yuxuanwu98@163.com (Y.W.)
[2] Key Laboratory of Ministry of Education on Pollution Control and Ecosystem Restoration in Industry Clusters, Guangzhou 510006, China
[3] Guangdong Provincial Key Laboratory of Solid Wastes Pollution Control and Recycling, Guangzhou 510006, China
* Correspondence: huayin@scut.edu.cn

Contents

Table S1. The main experimental drugs
Section S2. 16S rRNA sequencing specific processes

Table S1. The main experimental drugs.

	Drug	Manufacturer
1	Al $(NO_3)_3 \cdot 9H_2O$	Guangzhou Chemical Reagent Factory
2	Ca $(NO_3)_2 \cdot 4H_2O$	Sinopharm Chemical Reagent (Shanghai)
3	NaOH	Guangzhou Chemical Reagent Factory

Section S2. 16S rRNA sequencing specific processes

16S rRNA sequencing specific processes：Shenzhen Micro Science Alliance Technology Group Co., Ltd is responsible for completing the work of DNA extraction, PCR amplification, library construction, and sequencing, as well as data analysis, in which the sample DNA was extracted using the CTAB method, The PCR product was recovered using the Universal DNA (TianGen, China) Purification and Recovery Kit. The library was constructed using the NEB Next® Ultra DNA Library Prep Kit, and the constructed library was detected and quantified by Q-PCR using Agilent 5400. After the library was qualified, it was sequenced using the Illumina sequencing platform.
